# Fibrinogen/albumin ratio index is an independent predictor of recurrence-free survival in patients with intrahepatic cholangiocarcinoma following surgical resection

**DOI:** 10.1186/s12957-021-02330-2

**Published:** 2021-07-20

**Authors:** Hu Liu, Guoteng Qiu, Fengjuan Hu, Hong Wu

**Affiliations:** 1 Department of Liver Surgery, Liver Transplantation Division, West China Hospital, Sichuan University, Chengdu, 610041 China; 2 Laboratory of Liver Surgery, West China Hospital, Sichuan University, Chengdu, 610041 China; 3 The Center of Gerontology and Geriatrics, West China Hospital, Sichuan University, Chengdu, 610041 China

**Keywords:** Intrahepatic cholangiocarcinoma, Fibrinogen, Albumin, Fibrinogen-to-albumin ratio, Prognosis, Surgery

## Abstract

**Background:**

Systemic inflammation and nutritional status are associated with tumor development and progression. This study investigated the prognostic value of fibrinogen/albumin ratio index (FARI) in predicting recurrence-free survival (RFS) in patients with intrahepatic cholangiocarcinoma (ICC) undergoing hepatectomy.

**Methods:**

A retrospective cohort was conducted including patients who received curative hepatectomy for ICC at our hospital between May 2010 and December 2016. We collected the preoperative hematologic parameters and clinical data of all patients. Time-dependent receiver operating characteristic curve was used to identify the optimal cutoff value of FARI. The association between FARI-high and FARI-low group was investigated by using the Kaplan–Meier method. A nomogram based on the results of univariate and multivariate analysis was established.

**Results:**

A total of 394 patients with ICC who underwent hepatectomy at our hospital were enrolled. K-M analysis revealed that increased FARI was related to reduced RFS (P < 0.001). The multivariate analysis indicated that tumor number, tumor–node–metastasis stage, lymph node metastasis, cirrhosis, serum carbohydrate antigen 19-9, and FARI were independent predictors of RFS, and the ROC curve analysis showed that the optimal cutoff value for FARI was 0.084 based on the Youden index. The nomogram for FARI showed satisfactory accuracy in predicting RFS for ICC patients undergoing hepatectomy (C index = 0.663; AIC = 3081.07).

**Conclusion:**

Preoperative FARI is an independent predictor of RFS in patients undergoing hepatectomy for ICC, and the nomogram can be useful for clinical decision-making in the postoperative management of these patients.

**Supplementary Information:**

The online version contains supplementary material available at 10.1186/s12957-021-02330-2.

## Introduction

Hepatocellular carcinoma and intrahepatic cholangiocarcinoma account for ~ 85% of primary liver tumors. Intrahepatic cholangiocarcinoma (ICC), which originates in second-order bile ducts, accounts for 10–15% of hepatobiliary malignancies [1]. The incidence for ICC has been increasing in most areas of the world in recent years [2, 3]. The 5-year overall survival for ICC is ~ 20%; this low rate may be attributable to the aggressivity and heterogeneity of the tumors [4]. Surgery is considered the most effective treatment but only about 20% of patients undergo potentially curative resection [5]. Chemotherapy is the standard of care for patients with unresectable ICC [6, 7], while immunotherapy has recently shown favorable results, suggesting new possibilities in the treatment of ICC [8].

The American Joint Committee on Cancer (AJCC) tumor–node–metastasis (TNM) system is widely used for hepatobiliary cancer staging and can help clinicians to determine the best course of treatment for patients. However, the TNM system has some limitations, specifically with respect to the classification of T stage. One study showed that the risk of death was lower for TNM stage T3 than for stages T1b and T2 [9], while another reported comparable overall survival rates between stages T2 and T3 [10]. Based on these findings, many researchers have called for a new system for evaluating and predicting the outcome of ICC [11, 12].

In order to explore more clinicopathological features that may be related to tumor prognosis, researchers are committed to studying various indicators, such as systemic inflammation, morphology, immunohistochemistry, and surgical methods [13–17]. Systemic inflammation plays a critical role in cancer development and progression. For example, the accumulation of neutrophils within a tumor area was found to be associated with a higher aggressivity of ICC [18], whereas a larger population of CD4+ and CD8+ lymphocytes was linked to better prognosis [18, 19]. Inflammation-relatedvariables have shown satisfactory predictive value [20–23], such as neutrophil-to-lymphocyte ratio (NLR), γ-glutamyltransferase-to-lymphocyte ratio (GLR), and albumin-to-alkaline phosphatase (ALP) ratio (AAPR). Poor nutritional status has also been linked to reduced survival rates in cancer [24, 25]. The fibrinogen/albumin ratio index (FARI) was recently established to evaluate long-term survival in cancer patients [26–29] based on the finding that a higher FARI was associated with worse prognosis. However, the value of FARI in predicting the outcome of ICC patients undergoing hepatectomy is unknown. In this study, we evaluated the prognostic value of FARI in these patients and established a nomogram for predicting recurrence-free survival (RFS) based on this index.

## Patients and methods

### Study population

The study enrolled 394 consecutive patients who underwent surgical resection for ICC between May 2010 and December 2016 at the West China Hospital. All patients were newly diagnosed with ICC based on histopathologic examination after surgery and were undergoing surgery for ICC for the first time. Patients’ medical records were analyzed, including the hematologic test results obtained closest to the date of surgery. The exclusion criteria were as follows: patients with other primary malignancies or extrahepatic metastasis; patients who had received transarterial chemoembolization, chemotherapy, radiofrequency ablation, or other types of anticancer therapy before surgical resection or who were treated with palliative surgery; and patients with missing clinicopathologic data. Informed consent was obtained from each patient or a relative prior to enrollment. The study was carried out in accordance with the Declaration of Helsinki [30] and the ethical guidelines for clinical studies of the West China Hospital, and the study protocol was reviewed and approved by the ethics committee of West China Hospital (No. 2014-37).

### Clinical data collection and follow-up

Patient records including preoperative hematologic parameters were retrieved from electronic or handwritten medical records at the West China Hospital. The following information was obtained for each patient: age; sex; ascites; cirrhosis; hepatitis B surface antigen (HBsAg); total bilirubin (TBIL); ALP; albumin; carbohydrate antigen 19-9 (CA19-9); tumor size and number; macrovascular and microvascular invasion; TNM stage; tumor differentiation; neutrophil, lymphocyte, and platelet counts; and fibrinogen level in peripheral blood. FARI, NLR, and GLR were calculated as follows: FARI = fibrinogen concentration (g/l)/albumin concentration (g/l); NLR = neutrophil count/lymphocyte count; and GLR = γ-glutamyltransferase concentration (U/l)/lymphocyte count (10^12^/l). AAPR was calculated by dividing serum ALB level (g/l) by serum ALP level (U/l).

The patients were regularly screened for recurrence by monitoring plasma levels of ICC-specific tumor markers and through contrast-enhanced computed tomography performed every 3 months in the first year after surgery, every 6 months in year 2, and annually thereafter [31]. Full blood count and biochemistry, liver function, and other laboratory tests were performed at each visit. Follow-up treatment for patients who experienced recurrence was not recorded. The primary endpoint was RFS, defined as the time from surgery to the date of first recurrence or death. Patients without the above-described events were censored at the last follow-up (in December 2018).

### Statistical analysis

Statistical analyses were performed using SPSS v26.0 software (SPSS Inc., Chicago, IL, USA) and Prism v8.0 software (GraphPad, La Jolla, CA, USA). Categorical variables were compared using Pearson’s chi square test or Fisher’s exact test, as appropriate. Continuous data were compared using the Mann–Whitney U test or Student’s t test. To calculate the optimal cutoff values of FARI, NLR, GLR, and AAPR for predicting 5-year RFS, we generated receiver operating characteristic (ROC) curves and selected the maximum Youden index as the cutoff [32]. The area under the ROC curve (AUC) provided a measure of overall performance of hematologic markers. We used the cutoff value of FARI to divide the cohort into 2 groups (FARI-high and -low) and compared their baseline characteristics. Survival curves were generated with the Kaplan–Meier method and were compared with the log-rank test. Variables with a *P* value < 0.2 in the univariate Cox proportional hazards regression analysis were included in the multivariate analysis; a nomogram was constructed based on the results [33] using R v4.0.3 (https://www.r-project.org/) with 1000 bootstrap resamples. The performance of the nomogram was assessed based on concordance index (C index) and Akaike information criterion (AIC); these were also calculated for other variables. A larger C index indicates a more accurate prediction [34] while a lower AIC indicates less loss of information and is more representative of the variable [35]. A 2-sided P < 0.05 was considered statistically significant.

## Results

### Characteristics of the study population

The clinicopathologic characteristics of the patients are summarized in Table 1. Of the 394 patients, 191 (48.5%) were male; the median age was 59 years (interquartile range, 50–65 years), and 94 (23.9%) were positive for HBsAg. According to the AJCC staging manual (8th edition), tumor size was ≤ 5 cm in 148 (37.6%) cases and > 5 cm in 246 (62.4%) cases. The median tumor size in the whole cohort was 5.9 cm. Most cases (291, 73.9%) had a solitary tumor. A total of 281 patients (71.3%) had increased serum CA19-9 (reference value, 22 U/ml). Based on the postoperative pathology examination, 102 samples (25.9%) were lymph node-positive, 60 (15.2%) showed macrovascular invasion, and 46 (11.7%) showed microvascular invasion. Cirrhosis and ascites were observed in 38 (9.6%) and 51 (12.9%) patients, respectively.

**Table 1:** Relationship between demographic and clinicopathologic characteristics and FARI in patients with intrahepatic cholangiocarcinoma

Characteristic	Total (*n* = 394)	FARI-high (*n* = 168)	FARI-low (*n* = 226)	P value
Age in years, median (IQR)	59 (50–65)	60 (52–65)	58 (49–65.8)	0.577
Male sex	191 (48.5)	82 (48.8)	109 (48.2)	0.909
HBsAg positive	94 (23.9)	35 (20.8)	59 (26.1)	0.206
Ascites	51 (12.9)	25 (14.9)	26 (11.5)	0.323
TBIL [μmol/l], mean (SD)	19.2 (39.3)	24.0 (57.7)	15.6 (14.1)	0.022
ALP [U/l], mean (SD)	148.7 (142.2)	191.0 (185.2)	117.3 (87.1)	< 0.001
ALB [g/l], mean (SD)	42.1 (4.3)	39.9 (4.5)	43.7 (3.3)	< 0.001
CA19-9				
< 22	113 (28.7)	38 (25.6)	75 (33.2)	0.022
≥ 22	281 (71.3)	130 (74.4)	151 (66.8)	
Tumor size				
≤ 5 cm	148 (37.6)	42 (25)	89 (39.4)	0.003
> 5 cm	246 (62.4)	126 (75)	137 (60.6)	
Solitary tumor	291 (73.9)	117 (69.6)	174 (76.9)	0.533
Macrovascular invasion	60 (15.2)	30 (17.9)	30 (17.9)	0.211
Microvascular invasion	46 (11.7)	19 (11.3)	27 (16.1)	0.846
Cirrhosis	38 (9.6)	17 (10.1)	21 (12.5)	0.735
Tumor differentiation				
Poor	287 (72.8)	129 (76.8)	158 (69.9)	0.129
Moderate/well	107 (27.2)	39 (23.2)	68 (30.1)	
NLR				
High	268 (68.0)	141 (62.4)	127 (75.6)	< 0.001
Low	126 (31.9)	27 (11.9)	99 (58.9)	
GLR				
High	191 (48.5)	100 (44.2)	91 (54.2)	< 0.001
Low	203 (51.5)	68 (30.1)	135 (80.4)	
AAPR				
High	169 (42.9)	32 (12.2)	137 (81.5)	< 0.001
Low	225 (57.1)	136 (60.2)	89 (52.9)	

Data are presented as n (%) unless otherwise indicated

Abbreviations: *AAPR* albumin-to-alkaline phosphatase ratio, *ALB* albumin, *ALP* alkaline phosphatase, *CA19-9* carbohydrate antigen 19-9, *FARI* fibrinogen/albumin ratio index, *GLR* γ-glutamyltransferase-to-lymphocyte ratio, *HBsAg* hepatitis B surface antigen, *IQR* interquartile range, *NLR* neutrophil-to-lymphocyte ratio, *SD* standard deviation, *TBIL* total bilirubin

**Fig. 1:**
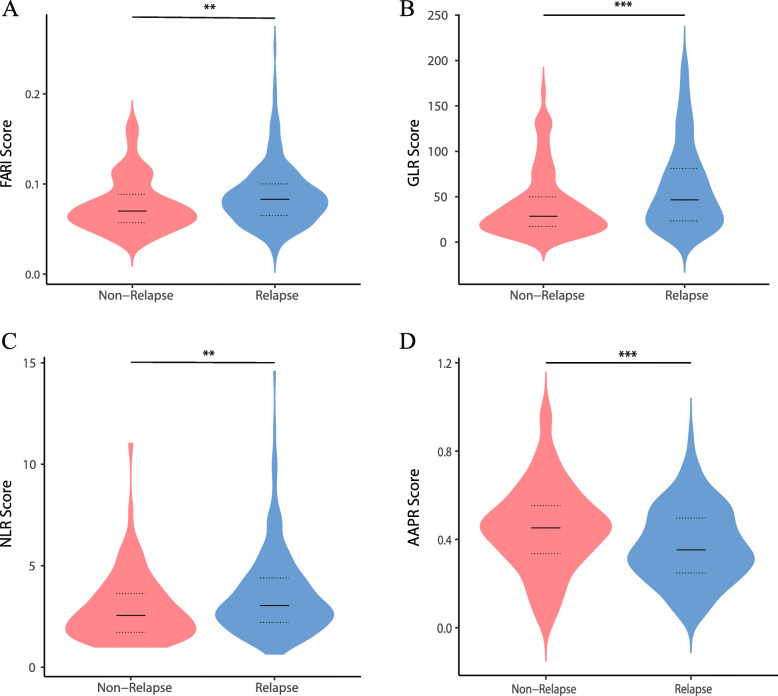
Scores for different parameters used to predict RFS in patients with ICC. Violin plots showing the distribution of FARI (**A**), GLR (**B**), NLR (**C**), and AAPR score (**D**) in non-relapsed and relapsed groups at the end of follow-up. Solid lines represent the median value; dotted lines represent quartiles. *P < 0.05, **P < 0.01, ***P < 0.001

We determined the optimal cutoff values of biochemical indices for estimating RFS by plotting ROC curves; the values were 0.084 for FARI, 2.30 for NLR, 41.41 for GLR, and 0.42 for AAPR, with AUCs of 0.606 (*P* = 0.001), 0.611 (*P* = 0.001), 0.621 (*P* < 0.001), and 0.621 (*P* < 0.001), respectively (Supplementary Fig. 1). Based on the cutoff value of FARI, we divided the cohort into 2 groups: 168 patients with FARI value > 0.084 were classified as FARI-high, whereas 268 were stratified into the FARI-low group. Relapsed patients had higher FARI, GLR, and NLR and lower AAPR than non-relapsed patients (Fig. 1). FARI was weakly correlated with NLR (r = 0.242, P < 0.001) and GLR (r = 0.235, *P* < 0.001) and showed a weakly but statistically significant negative correlation with AAPR (r = − 0.474, *P* < 0.001).

**Fig. 2:**
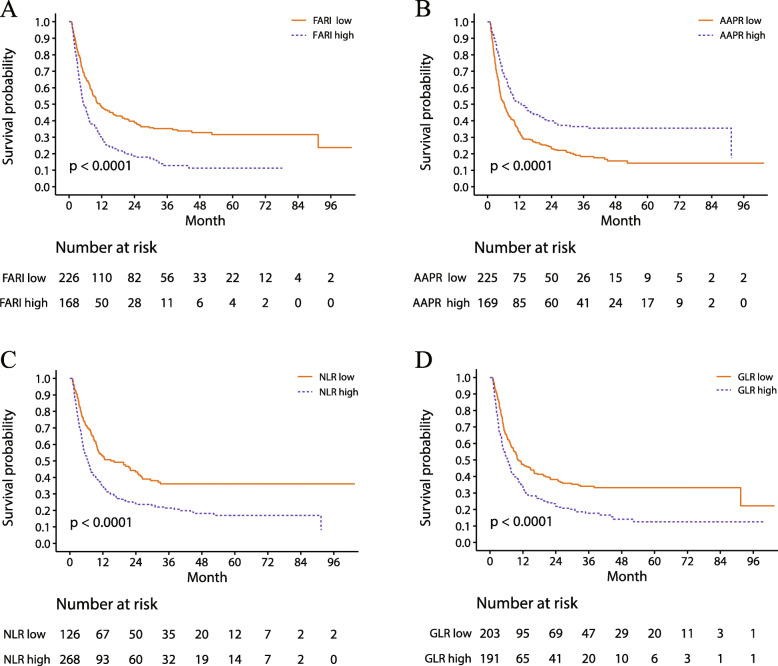
Survival analysis of patients with ICC according to different variables. Kaplan–Meier survival curves of RFS in groups defined by cutoff values of FARI (**A**), GLR (**B**), NLR (**C**), and AAPR score (**D**)

Of the 394 patients, 292 relapsed during the study period. The 1-, 3-, and 5-year overall RFS was 40.6%, 26.1%, and 23.3%, respectively. The Kaplan–Meier analysis with log-rank test showed that increased FARI, NLR, and GLR and decreased AAPR were associated with worse prognosis (Fig. 2). On the other hand, patients with microvascular invasion, cirrhosis, poor tumor differentiation, tumor size > 5 cm, TNM stage > T2, multiple tumors, or elevated CA19-9 level had lower RFS (Supplementary Fig. 2).

In the univariate Cox regression analysis, 13 clinicopathologic variables were potentially related to RFS (*P* < 0.2) and were analyzed with a multivariate regression model; tumor number, TNM stage, lymph node metastasis, cirrhosis, serum CA19-9, and FARI were identified as independent risk factors for tumor recurrence (Table 2).

**Table 2:** Univariate and multivariate analyses of prognostic factors for recurrence-free survival in patients with intrahepatic cholangiocarcinoma

Variable	Univariate analysis			Multivariate analysis		
	HR	95% CI	*P* value	HR	95% CI	*P* value
Age ≥ 60	0.983	0.781–1.237	0.882			
Male sex	1.157	0.919–1.456	0.215			
HBsAg	1.151	0.886–1.495	0.293			
Ascites	1.561	1.135–2.146	**0.006**			
Solitary tumor	1.564	1.218–2.009	**< 0.001**	1.308	1.006–1.701	**0.045**
Tumor size ≥ 5 cm	1.364	1.062–1.751	**0.015**			
Macrovascular invasion	1.147	0.835–1.577	0.397			
Microvascular invasion	1.774	1.273–2.473	**0.001**			
Lymph node metastasis	2.516	1.958–3.234	**< 0.001**	1.918	1.444–2.548	**< 0.001**
Cirrhosis	1.707	1.202–2.424	**0.003**	1.715	1.190–2.472	**0.004**
CA19-9 ≥ 22	1.770	1.350–2.322	**< 0.001**	1.606	1.214–2.123	**0.001**
Liver capsule invasion	1.310	1.016–1.688	**0.037**			
TNM > T2	1.724	1.292–2.300	**< 0.001**	1.732	1.042–2.879	**0.034**
FARI			**< 0.001**			**0.016**
Low	Ref					
High	1.790	1.419–2.257		1.355	1.058–1.737	
NLR			**< 0.001**			
Low	Ref					
High	1.714	1.322–2.221				
GLR			**< 0.001**			
Low	Ref					
High	1.627	1.291–2.050				
AAPR			**< 0.001**			
High	Ref					
Low	1.695	1.335–2.152				

P values in boldface are statistically significant (P < 0.05)

Abbreviations: *AAPR* albumin-to-alkaline phosphatase ratio, *CA19-9* carbohydrate antigen 19-9, *FARI* fibrinogen/albumin ratio index, *GLR* γ-glutamyltransferase-to-lymphocyte ratio, *HBsAg* hepatitis B surface antigen, *HR* hazard ratio, *NLR* neutrophil-to-lymphocyte ratio, *Ref* reference, *TNM* tumor–node–metastasis stage

### Prognostic nomogram for RFS

**Fig. 3:**
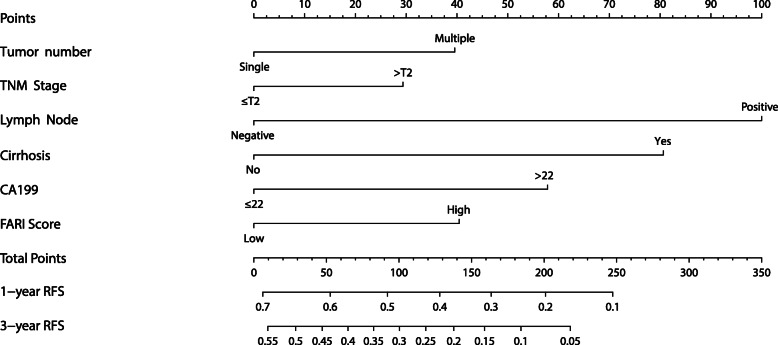
Nomogram for predicting the probability of 1- and 3-year RFS in ICC patients undergoing hepatectomy

A nomogram for predicting RFS was constructed based on the following independent risk factors: tumor number (solitary vs multiple), TNM stage (≤ T2 vs > T2), lymph node metastasis (yes vs no), cirrhosis (yes vs no), serum CA19-9 (≤ 22 or > 22), and FARI (high vs low) (Fig. 3). Each factor was associated with a score, the sum of which predicted the 1- and 3-year recurrence probability of patients. The calibration plot showed satisfactory consistency between the nomogram-predicted RFS and actual survival outcomes (Supplementary Fig. 3). Furthermore, the discriminatory capacity of the nomogram and other parameters was evaluated by comparing C index and AIC values (Table 3). The C index of the nomogram was 0.663 (95% confidence interval, 0.630–0.696), which was higher than the C indices of NLR (0.654), GLR (0.652), and AAPR (0.655). These results indicate that FARI better predicts RFS than these other variables.

**Table 3:** Predictive value of the FARI-based nomogram and 3 variables for recurrence-free survival in patients with intrahepatic cholangiocarcinoma

Parameter	C index	95% CI	*P* value	AIC
FARI	0.663	0.630–0.696	< 0.001	3081.07
NLR	0.654	0.622–0.686	< 0.001	3079.11
GLR	0.652	0.619–0.685	< 0.001	3079.93
AAPR	0.655	0.622–0.688	< 0.001	3082.09

Abbreviations: *AAPR* albumin-to-alkaline phosphatase ratio, *AIC* Akaike information criterion, *CI* confidence interval, *FARI* fibrinogen/albumin ratio index, *GLR* γ-glutamyltransferase-to-lymphocyte ratio, *NLR* neutrophil-to-lymphocyte ratio

## Discussion

ICC has poor prognosis because of the aggressivity and heterogeneity of the tumors. As the current AJCC TNM staging system is inadequate for prognostic evaluation of ICC patients who undergo hepatectomy, better tools that can guide clinical decision-making are needed.

Noninvasive markers such as the standard inflammatory indicators NLR, GLR, and AAPR have been used to monitor the progression and predict the outcome of malignant tumors [20–22]. These can easily be calculated by obtaining neutrophil and lymphocyte counts or by measuring γ-glutamyltransferase, serum albumin, and ALP levels. Neutrophil transmigration across the epithelium causes the disruption of epithelial adherens junctions through the release of elastase [36], which can promote tumor progression. Lymphocytes are associated with self-immunity, and patients with malignant tumors are often in a state of immunosuppression that promotes tumor growth, invasion, and metastasis [37, 38]. Immunologic markers are influenced by infection and bone marrow suppression induced by chemotherapy, radiotherapy, or other factors, which reduces their predictive value. As a biochemical marker, FARI is more stable in the circulation and has been used to evaluate long-term prognosis in various cancers including pancreatic ductal adenocarcinoma [39], gastrointestinal stromal tumors [26], and colorectal liver metastases [27]. In this study, we evaluated the prognostic value of FARI for ICC. The optimal cutoff value for FARI in the ROC curve analysis was 0.084, which diverges from the value of 0.076 reported for colorectal liver metastases [26] and the value of 0.08 in gallbladder cancer [28]. The reason for this discrepancy in the cutoff value among tumor types is unclear, but it may be attributable to differences in the biological characteristics of each tumor.

We divided the cohort into FARI-high and -low groups according to the cutoff value of FARI. Patients in the former group had a larger tumor size, elevated serum CA19-9, lymph node metastasis, and elevated TBIL and ALP levels, suggesting that FARI can reflect disease progression and metastasis in ICC. We also found that a high FARI was correlated with inflammatory indicators including high NLR and GLR and low AAPR. Moreover, the survival analysis revealed that RFS was shorter in ICC patients with a high FARI than in those with a low FARI. A nomogram was constructed based on 6 independent prognostic factors that significantly influenced RFS in the univariate and multivariate analyses, including tumor number, TNM stage, lymph node metastasis, cirrhosis, serum CA19-9, and FARI score. In our assessment of nomogram performance in predicting RFS, the C index and AIC were 0.663 and 3081.07, respectively, which were higher than the values for inflammatory indicators. Thus, FARI may be useful for predicting the clinical outcome of patients with ICC undergoing hepatectomy.

Fibrinogen is a large fibrous glycoprotein produced by hepatocytes that has been implicated in cancer growth and metastasis [39]. It is frequently detected in tumors and contributes to the formation of tumor-reactive stroma; moreover, fibrogen can promote tumor angiogenesis by binding several growth factors including fibroblast growth factor 2 (FGF-2) and vascular endothelial growth factor (VEGF), leading to tumor progression [40, 41]. Fibrinogen was shown to promote the malignant transformation of tumors by inducing epithelial-to-mesenchymal transition via the mammalian target of rapamycin (mTOR)/protein kinase B (AKT) signaling pathway [42], and inhibited the cytotoxic activities of natural killer cells in tumors [43]. It has been found to be a key marker in progression of colon cancer [44]. Thus, fibrinogen is a useful marker for monitoring cancer progression.

Albumin is the most abundant protein in human serum and reflects the biosynthetic function of the liver and as well as the preoperative nutritional status of patients. Malnutrition is frequently observed and is related to outcome in various malignancies. Cancer-related malnutrition is associated with impaired immune function and increased proinflammatory cytokine levels, and some studies have shown that lower preoperative albumin level was associated with poor prognosis [45–47]. All of these factors contribute to low survival in cancer patients [48]. Thus, albumin level is also a useful marker for evaluating clinical outcomes in cancer [49].

There were some limitations to our study. First, the single-center retrospective design may have introduced a bias in our analyses. Second, our sample size was small and there was no external validation of our results. Third, the C index of the nomogram was not ideal, as a high predictive value is not equivalent to clinical applicability; thus, our nomogram needs to be improved by including other clinical variables. In addition, compared with existing biomarkers, the AUC of FARI is relatively low, so the application value of FARI might be limited. Although excellent performance had been found in tumors [26–29], well-designed researches are still needed to explore the applicability in I CC. Finally, we did not investigate the mechanisms by which fibrinogen and albumin influence ICC recurrence. Further studies using animal models are needed in order to address this point.

## Conclusion

The results of this study demonstrate that preoperative FARI is an independent prognostic factor for RFS in patient with ICC undergoing hepatectomy. A high FARI was associated with elevated risk of postoperative recurrence, and a nomogram that included FARI was a better predictor of recurrence than NLR, GLR, and AAPR. Thus, FARI is an objective, low-cost, easily measurable, and noninvasive prognostic indicator that can aid clinical decision-making in the management of ICC following hepatectomy.

## Supplementary Information

**Additional file 1: Supplementary Figure 1.** Prognostic value of FARI, GLR, NLR, and AAPR for RFS in ICC patients evaluated by ROC curve analysis. (EPS 569 kb)

**Additional file 2: Supplementary Figure 2.** Survival analysis of patients with ICC according to clinicopathologic variables. (A–G) Kaplan–Meier survival curves of RFS in groups defined by cirrhosis (A), MVI (B), differentiation (C), TNM stage (D), tumor size (E), CA-199 (F), and tumor number (G). (EPS 3398 kb)

**Additional file 3: Supplementary Figure 3.** Calibration plot of nomogram-predicted3-year RFS in patients with ICC. (EPS 620 kb)

## Data Availability

The analyzed data sets generated during the study are available from the corresponding author on reasonable request.
